# Providing ramps during lay has larger impacts on laying hens than ramps at rearing

**DOI:** 10.1016/j.psj.2024.104101

**Published:** 2024-07-31

**Authors:** M.J. Toscano, A.S. Jalali, J.M. Siegford, A. Stratmann

**Affiliations:** ⁎ Center for Proper Housing: Poultry and Rabbits (ZTHZ), Division of Animal Welfare, VPH-Institute, University of Bern, Zollikofen, Switzerland; † Department of Animal Science, Michigan State University, East Lansing, MI, USA; ‡ Commercial Farm Preventative Veterinary Care Service, Sari, Iran

**Keywords:** bone health, movement, feather quality

## Abstract

Commercial laying hen housing is shifting from traditional cages to non-cage housing systems, such as the aviary, which has gained popularity due to potential for more species-typical behavior. However, birds housed in aviaries may have difficulties moving through the vertical tiers of the system leading to health problems such as keel bone fracture (**KBF**). One possible way to improve movement is to add ramps into an aviary system, allowing hens to walk between tiers rather than jump or fly. The objective of this study was to evaluate the impact of adding ramps to rearing and laying aviaries on bird health, production, and movement across vertical tiers of the aviary. Lohmann Selected Leghorn pullets were raised in 2 treatments: 4 pens (600 birds/pen) were raised with wire mesh ramps to aid movement between aviary tiers (**RR**) and 4 pens (600 birds/pen) were raised without ramps (RO). At 17 wk of age (**WOA**), birds were moved to the laying facility, in which 16 aviary pens with 225 birds/pen were populated. Half the pens (n = 8) were supplemented with wire mesh ramps (**LR**) and the other half were not (**LO**). Within each laying treatment group, 4 pens were populated with RR hens and 4 pens were populated with RO hens, creating 4 treatment combinations (RRLR, RRLO, ROLR, ROLO). From each pen, 15 focal hens were selected for radiographic imaging of their keel bones taken at 21, 36, 45, and 60 WOA and the images were subsequently scored for KBF severity. Focal hens were also scored for feather condition and footpad quality at 36 and 60 WOA using a standardized welfare assessment protocol. The number of downward transitions among aviary areas and falls were recorded at 19 to 20 and 30 to 31 WOA. Data were analyzed using (generalized) linear mixed models in R software. When ramps were available, they were used in most of the observed downward transitions (79% in ROLR and 86% in RRLR). Hens who received ramps in lay (i.e., RRLR and ROLR) showed more transitions immediately after lights on compared to midday or dusk phases (*p* < 0.001), performed more transitions from the first aviary tier compared to nest or top tier (*p* = 0.013) and had lower KBF severity than those who did not receive ramps in the laying aviaries (ROLO, RRLO; *p* < 0.001). At 60 WOA, hens in the RRLR treatment had greater feather coverage than those in ROLR and RRLO treatments (*p* < 0.001). Birds in the RRLR treatment had better foot health overall than those in treatments without ramps in lay (*p* = 0.018). Providing ramps to hens in aviaries appeared to be the preferred means of transitioning between aviary tiers though had positive effects on welfare parameters such as food health, feather coverage, and KBF severity, without negative impacts on production. Benefits were seen primarily when ramps were provided in lay, though their installation in rearing provided evidence of easier adaptation to the laying barn. Our study supports providing ramps throughout the lifetime of the bird to accommodate hens’ preferred means of moving vertically in aviaries and deliver consequent benefits to health and welfare.

## INTRODUCTION

The housing of laying hens is a major interest for the public and stakeholders in light of increasingly negative perception of traditional cages in the US, Europe, and across the world. As a consequence of this developing perspective, the need for noncage housing systems that are viable in terms of hen welfare, production, and management has become critical. Aviaries, defined as multitier structures within a barn characterized by several vertically stacked tiers containing different resources such as feed, water, perches, and nests distributed among these tiers as well as a litter-covered floor, are one particular option that is considered a viable alternative.

Aviaries provide many benefits that are believed to lead to improved welfare, most importantly the ability to perform a greater repertoire of highly motivated behaviors such as dust-bathing, use of nests, and roosting at elevated positions (Weeks and Nicol, 2006; Lay et al., 2011). While a major benefit of aviaries is that they offer the resources to allow behaviors associated with better welfare, the birds are required to move throughout the system to access those resources. For instance, dust-bathing is typically performed in the litter on the floor of the system whereas birds prefer to roost preferentially at greater heights, and nests are typically located in a mid-tier position. The various vertical tiers are mostly reachable via jumping and flying between perches and or platforms positioned at varying distances and angles. Thus, while an aviary contains many of the resources to achieve a relatively high quality of life, pullets must develop the capacity to access those resources and then maintain them through the production periods.

In order for hens to move effectively, pullets must specifically develop in terms of musculo-skeletal properties (Casey-Trott et al., 2017; Rufener and Toscano, 2020) and spatial-cognitive abilities (Freire, 2020). Mechanical loading and load-bearing exercises such as running and wing flapping both affect bone formation and alter bone characteristics in ways that improve skeletal integrity (e.g., make bones stronger) (Rufener and Toscano, 2020). Exposure to elevated heights during rearing can allow birds to gain the navigation skills to judge distances and required speeds to move up inclines (e.g., improve spatial cognitive development) (Kozak et al., 2016a,b; Pullin et al., 2024). Comparisons during the laying period between floor and aviary reared birds reveal that birds reared in aviaries performed more vertical movements and had better overall production (Michel and Huonnic, 2003; Colson et al., 2008). In summary, these studies demonstrate the importance of adequate development during the rearing period to facilitate transfer to and performance in aviaries during the laying period.

In contrast, poor development during the rearing period likely has far-reaching, negative consequences for hens and how they use their environment. For instance, hens lacking exposure to different heights were found to lay a greater proportion of eggs on the floor rather than in raised nest boxes which the authors reasoned was due to difficulty in accessing the higher platform and nest boxes (Appleby et al., 1983, 1988; Colson et al., 2008). Hens and pullets desiring to move from one vertical tier to another can show a variety of behaviors indicative of hesitation, frustration, or may end up not making the transition at all (Pettersson et al., 2017; Norman et al., 2018). Likely related to these navigation problems and the combined effects of improper bone development are keel bone fractures, one of the greatest welfare problems facing the commercial laying hen industry (Harlander-Matauschek et al., 2015). Although the precise cause of injury is unknown, previous investigations have suggested that locomotion within the aviary (Stratmann et al., 2015a) or specific arrangement and positions of internal structures that likely affect movement (Richards et al., 2011a,b; Heerkens et al., 2015a,b; Stratmann et al., 2015b) are contributory factors to the injuries observed (for a review of explanations, see Toscano et al., 2020).

Multiple efforts have investigated the use of ramps to facilitate movement between aviary tiers to improve laying hens’ access to resources in these complex housing systems. Stratmann et al. (2015a) investigated whether ramps facilitate inter-tier movement and found that ramps were associated with reduced incidence of falls, collisions, and keel bone fractures, a finding supported by others (aviary systems: Heerkens et al. (2015b); single tier system: Pettersson et al. (2017)). Given the relatively poor flight abilities of laying hens (compared to smaller, more agile bird species; see Tobalske, 2015), the facilitation of walking by providing ramps for movement between tiers is likely a safer mode of locomotion (vs. flying behavior) within the confined conditions of aviaries.

More recent efforts have begun exposing chicks and pullets to ramps (Norman et al., 2018, 2019, 2021; Stratmann et al., 2022) where perceived benefits are likely to be encouraged load-bearing locomotion and cognitive development (Kozak et al., 2016a,b). In a review, Harlander-Matauschek et al. (2015) suggested that providing ramps to young chicks could promote wing-assisted incline running, and thus improve development of the keel bone and muscles as well as balancing abilities. Under experimental conditions, chicks reared with ramps were more successful at learning to move up ramps for a food reward, took less time to move up the ramp, and showed less hesitancy before transitioning (Norman et al., 2018). Stratmann et al. (2022) demonstrated that the majority of downward transitions during rearing were made with ramps; even at 14 weeks of age when pullets could easily fly or jump down, ramps were the preferred means for locomotion.

Taken together, optimizing the long-term health and welfare of laying hens within aviaries requires appropriate development during rearing and maintaining safe access to resources in adulthood, for which ramps may provide these benefits. However, despite the knowledge gained to date, it is unclear how combining ramps in rearing and laying phases can be optimized in terms of facilitating bird movement and access to resources, as well as overall health including bone fractures. To provide this information, the current study investigated whether encouraging greater and earlier locomotion among vertical tiers by providing ramps at rearing and/or laying would impact hen behavior, health, and productivity in the laying period. We hypothesized that pullets/hens with ramps would show greater movement within the aviary, superior health, and improved production (e.g., more nest laid eggs due to better use of nests resulting from improved ability to access nests).

## MATERIALS AND METHODS

### Ethics

All research protocols were approved by the Veterinary Office of the Canton of Bern (approval number BE55/17) and the Michigan State University Institutional Animal Care and Use Committee prior to the start of data collection.

### Animals and Housing During Rearing

A total of 4,800 Lohmann Selected Leghorn (**LSL**) day-old, non-beak trimmed chicks were supplied by a commercial hatchery and reared on site in eight pens of a rearing barn with 600 chicks per pen. Rearing pens were equipped with either of 2 multi-tier aviary systems differing in structure: 4 pens contained an aviary structure with vertical tiers stacked directly on one another (Direct: Inauen Natura, Inauen AG, Appenzell, Switzerland) whereas the remaining 4 pens contained an aviary with tiers stacked in an offset configuration (Offset: Landmeco Harmony, Globogal AG, Lenzburg, Switzerland). For the purposes of the current study, in 2 rearing pens per aviary structure, the aviaries were equipped with ramps connecting the different tiers, which allowed birds to walk between tiers. Ramps had a width of 24 cm with a mesh size of 2 × 2 cm and varied in length depending on the aviary structure and position within the aviary. Thus, 4 rearing groups existed: 2 aviary structures (Direct & Offset) x 2 ramp treatments (Reared with ramps (**RR**) & Reared without ramps (**RO**)) with 2 pens per rearing group (detailed description of ramps during rearing phase are provided in Stratmann et al., 2022). The inclusion of 2 rearing houses is an artefact of our site and was not intended to be a key factor of interest but is required to ensure enough birds are in the laying barn. Previous work has compared the 2 rearing barns and provided a descriptive comparison of transitions between areas and pullet distribution (Stratmann et al., 2022). The chicks began on the lowest tier of the aviary with the floor covered in chick paper. Pullets were given a standard diet with a starter diet from one to eight WOA followed by a pullet feed from nine to 17 WOA. Artificial light was provided depending on WOA according to the LSL standard rearing procedure with light hours decreasing from 16 h to a minimum of 8 h at 12 WOA and increasing again to 10 h from 16 to 18 WOA. Additionally, daylight was provided through 2 window shades per pen that were automatically opened depending on WOA (see above) and closed at 16:30 h. After 7 d of age, the first tier was opened, and birds had access to the whole pen, including the litter which was covered with wood shavings. In the offset aviaries, access to litter was facilitated by a grid platform on both sides that connected the litter and first tier, whereas in the direct aviaries, wooden bars were provided on both sides connecting the litter and first tier. These structures were provided in each pen until 4 WOA, after which they were removed. From 5 WOA until the end of the rearing phase, birds had daily access between 10:00 h and 16:00 h to a pen-specific winter garden (size varied between 15 and 21 m^2^) where the floor was also covered with wood shavings. Wire mesh fencing prevented mingling between, and each garden was equipped with 5 wooden perches.

### Animals and Housing During the Laying Phase

At 18 WOA, hens were moved to 16 identical experimental pens in a layer barn with each pen containing 225 hens (3,600 hens in total). The remaining birds either distributed among 4 additional pens in the layer barn (900 pullets) that were not part of the current experiment or sold commercially. Four experimental pens in the layer barn were populated with hens from 2 pens of the rearing barn of the same rearing group (i.e., aviary structure x ramp treatment). During the population process, all hens were given a pen-specific colored leg ring (Fieger AG, Tuttwil, Switzerland); focal hens (n = 15 hens per pen; 240 hens in total) were selected in a stratified manner and given a flexible legband (Roxan Developments Ltd, Selkirk, United Kingdom) with an individual identification number.

**Figure 1:**
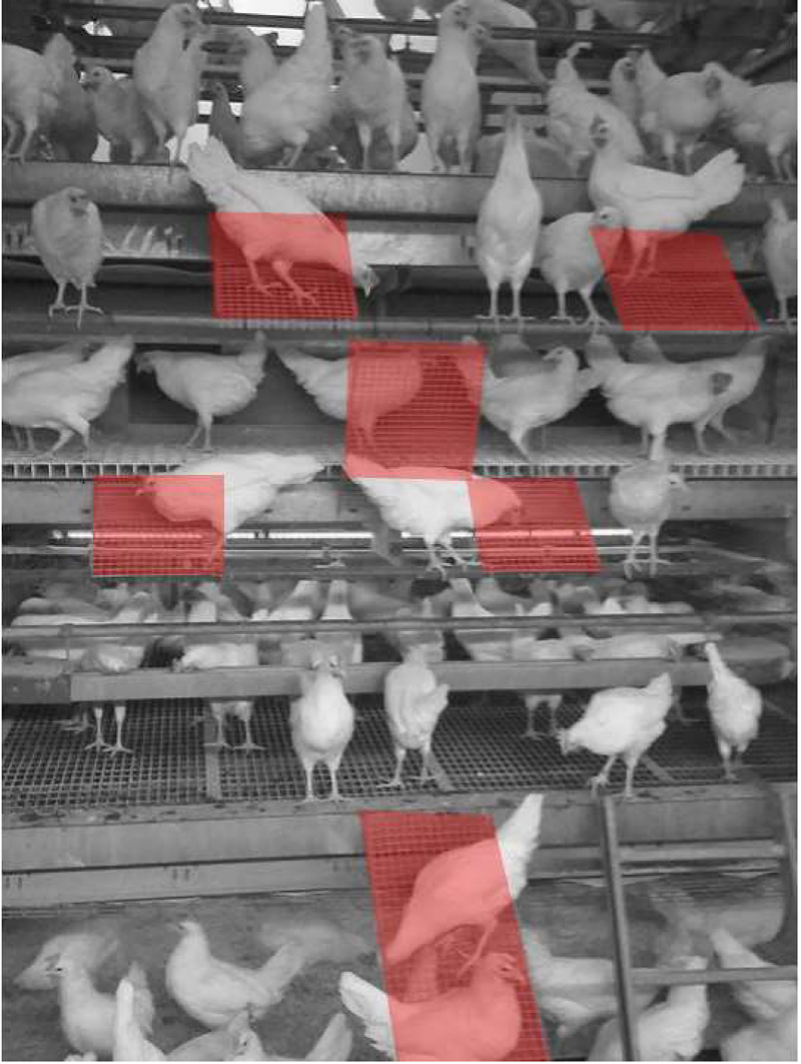
Aviary system (Bolegg Terrace, Vencomatic, Krieger AG, Ruswil, Switzerland) with 3 tiers used during the laying phase. In the laying ramp treatment (**LR**), ramps (highlighted in red) were installed to provide hens with a path among the vertical tiers in the aviary.

The layer barn (described previously in detail in Stratmann et al. (2015a)) was equipped with a commercial Bolegg Terrace aviary system with 3 tiers (Bolegg Terrace, Vencomatic, Krieger AG, Ruswil, Switzerland; Figure 1). Resources were provided on the different aviary tiers: feeding chains and nipple drinkers on the lower tier provided food and water; group nests provided on the middle tier. Food, water, and perches (diameter: 3.2 cm, length: 230 cm) were available on the top tier. Additional perches were installed on both sides of the aviary structure to facilitate movements between tiers. Animal density was 7.4 hens / m^2^ of accessible area (including all grid areas of the lower and upper tiers and littered floor area).

Artificial light was provided from 2:00 h until 17:00 h with a 5 min brightening phase in the morning (2:00 h–2:05 h) and a 30 min dimming phase (16:30 h–17:00 h) in the evening. Curtains in front of the windows were open from 8:00 h until 16:00 h to provide natural daylight. A winter garden (9.32 m^2^), containing wood shavings and a dust bathing area filled with sand, was accessed via pop holes that opened automatically at 10:00 h and were closed manually between 16:00 h and 16:30 h. The entire floor of each pen was covered with wood shavings that was resupplied approximately every 2 wk.

### Experimental Design

In half of the 16 pens, ramps were installed to connect the different tiers of the laying aviary whereas the remaining pens did not contain ramps (Laying aviary with ramps (**LR**) vs. Laying aviary without ramps (**LO**); n = 8 pens per treatment group). Ramps were installed in a manner that provided a vertical path between the aviary tiers to allow birds to walk between the tiers instead of jumping or flying (see Figure 1). Pullets were allocated to the different pens in the layer barn based on having access to ramps during the rearing phase (i.e., RR vs. RO) resulting in 4 treatment combinations: RRLR, RRLO, ROLR, and ROLO with 4 pens per treatment combination. Aviary structure during the rearing phase (i.e., offset vs. direct) was included as a factor in determining the distribution of pullets across the pens in the laying barn so that rearing barn was represented equally in the laying barn, but was not explicitly analyzed further. Radiographs of keels, body weights, and assessment of foot health and plumage were performed on focal birds as described below allowing for a detailed longitudinal examination.

### Data Collection

***Behavior.*** To evaluate potential benefits of ramp provision on behavior, videos were recorded using 2 infrared cameras per pen (Samsung SNO-6083R, Samsung Techwin CO., South Korea) and customized recording software (Multieye Green Watch, Recorder Version 2.4.2, Artec Technologies AG, Diepholz, Germany). Cameras were positioned on both sides of the aviary to provide coverage of the entire aviary height and width. Recordings were conducted on one day each at 2 different ages (i.e., 19–20; 30–31) during the laying phase. Due to constraints with the number of cameras, pens within each age were further split in 2 groups (A and B) with pens assigned in an alternating fashion. Within each age, videos were recorded in group A and B consecutively, resulting in 4 observed ages (WOA 19/20 and WOA 30/31). As we did not expect to find differences between the 2 age ranges, the 4 WOA were grouped resulting in a 2-level factor (i.e., time point 1 = WOA 19/20 and time point 2 = WOA 30/31). Hereafter, we refer to these time points within age as 19/20 and 30/31 WOA. At each observation age, the number of downward transitions between the different tiers of the aviary system and the number of falls were counted. Behaviors were counted at three times of day (**TOD** i.e., after lights on (including the brightening period), at midday, and during the dusk phase). At each TOD, 10 min of video were watched continuously and the number of observed downward transitions and the number of falls were counted (a total amount 5,760 min of video watched). For each transition, the start and end locations of the transition, as well as whether a ramp was used for the transition was recorded. The areas between which downward transitions were considered included: litter area, first (including first tier and associated perches), middle (including nest platforms and associated perches), and top tiers (including top tier and associated perches). In terms of ramp use, it was distinguished whether the hen used a ramp (ramp transition) or jumped/flew (non-ramp transition) directly from one tier to another. Ramp transitions were defined as a hen traveling either all or part of the ramp length to move between tiers, while a non-ramp transition was defined as hen moving between tiers without ever touching a ramp. Our study is limited to downward transitions only (vs. including upward as well) which was based on logistical challenges of collecting adequate quantities of observations. Downward movements were chosen as we believe they are more difficult compared to moving upwards (Scott et al., 1997). Falls were defined according to Stratmann et al. (2015a) where a fall was defined as a bird changing position in aviary tiers without having a successful landing phase and/or no signs of downward orientation behavior.

***Keel Bone Assessment***. Radiographic images of focal hens’ keels were collected at 21, 36, 45, and 60 WOA using a mobile radiograph unit (GIERTH HF 200 ML; radiograph tube Toshiba D-124 with maximal acceleration voltage of 100 kV; radiograph plate Canon CXDI-50G; software Canon CXDI Control Software NE) at a distance of 80 cm and voltage of 46 kV/2.4 mAs. To induce immobility during image capture, hens were hung upside down by both legs in padded metal shackles attached to a wooden frame using a procedure previously described by Rufener et al. (2018a) that minimizes distress while avoiding the need to anesthetize hens. The images were scored using a tagged, visual analog scale, that is generally seen as superior to traditional ordinal scales (Tuyttens et al., 2014). The specific scale used has been described (Rufener et al., 2018b) and is based on the amount of bone damaged and the severity of the fracture. Prior to scoring, images were coded to ensure the observer was blind to treatment.

***Plumage Condition****.* To assess the effect of providing ramps during the rearing and laying phases on plumage condition during the layer phase, feather damage of the focal hens was assessed at 2 different ages (i.e., WOA 36 and 60) using the scale developed by Tauson et al. (1984). The pictures of white laying hens from Welfare Quality (2009) were used as a reference, though the system was adapted so that poorer feather coverage was associated with a lower value and scored using a visual analog scale as with evaluations of keel fractures. For each body part (i.e., neck, chest, wings, tail, back and cloaca), a sheet with a 10 cm long visual analogue scale was superimposed on a diagram of the 4 scores represented on a linear line. A mark was placed on the visual analogue scale, and then later the distance of the mark from the origin was measured with a ruler. Scores of the different body parts were averaged for each hen at each WOA and the assessor was blind to treatment.

***Foot Health****.* To assess the effect of ramp provision on foot health, both feet of focal hens were assessed for pododermatitis, toe injuries, toe fractures, and occurrence of bumble foot at 2 different ages (i.e., WOA 36 and 60). Pododermatitis was assessed using a score from 0 to 2 with score 0 meaning the absence of any appearance and score 2 meaning severe pododermatitis (according to Welfare Quality). Toe wounds, fractures, and bumble foot were scored yes/ no per foot. Scores were averaged for both feet per hen and WOA and the assessor was blind to treatment.

***Body Mass****.* Body mass of focal hens was recorded during the assessment of plumage and foot condition at both WOA.

### Production

Production parameters collected from hens between 18 and 60 WOA included: egg-laying performance (%), feed consumption (kg), mortality (%), and floor eggs (%). Data was collected on a daily basis per pen and incorporated the number of live hens on that day, but then averaged in 4-wk periods as is common for Swiss production periods.

### Statistical Analyses

Data were analyzed using linear mixed-effects models (**LME**) and generalized linear mixed-effects models (**GLME**) in R (version 4.2.1, R Core Team, 2022) with RStudio as the user interface (version 4.2.1, Rstudio Team, 2022) applying the package lme4 (Bates et al., 2015). Model assumptions were checked visually using q-q plots for LME and the package DHARMa (Hartig, 2018) was used for general LME to check for a normal error distribution and homoscedasticity of the residuals. We used dummy variables with sum contrasts for tested factors and interactions. *P*-values were obtained by comparing the full model including all main effects and interactions to models each reduced by one main effect or interaction only. The model comparison was performed using a parametric bootstrap approach with the function “Pbmodcomp” from the “pbkrtest” package (Halekoh and Højsgaard, 2014). Model estimates and confidence intervals were calculated for the full models and displayed using the package “effects” (Fox, 2003). We did try to include rearing barn in the model as a random term, however our models did not converge, an outcome that is expected with our design of 4 pens for each rearing aviary type and 2 pens per treatment per aviary type, that is, n = 2.

In terms of behavioral data, the number of downward transitions was analyzed as a response variable including treatment (i.e., RRLR, RRLO, ROLR and ROLO), age (i.e., WOA 19/20 and 30/31) area (i.e., first, middle and top tier) and TOD (i.e., lights on, midday and dusk) as explanatory variables using LME. Pen was included as a random effect crossed with observation date and all interactions including the factor treatment were examined. The use of ramps for downward transitions was calculated as the percentage of the overall number of downward transitions observed in the pens where ramps were provided and analyzed descriptively.

The number of falls observed during video analysis were infrequent – in 414 out of 576 observations made throughout the video analysis, no falls were observed – thus the observed 162 falls were analyzed descriptively.

Regarding health data, keel bone fracture severity was analyzed as a continuous response variable with treatment (i.e., RRLR, RRLO, ROLR, and ROLO) and age (i.e., 21, 36, 45, and 60 WOA) as well as their interaction included as explanatory variables using LME. The average scores for plumage condition and pododermatitis were analyzed using LME and GLME, respectively, where the mean score for pododermatitis was analyzed with a binomial distribution (pododermatitis present / absent, score < 1). For both response variables, treatment (i.e., RRLR, RRLO, ROLR and ROLO), age (i.e., WOA 36 and 60), and their interaction were used as explanatory variables. The occurrences of toe injuries, toe fractures, and bumble foot were rare (toe fractures: 1.1%, toe wounds: 5.1%, bumble foot: 12%) and thus not analyzed. Body mass was analyzed using LME including the factors treatment (i.e., RRLR, RRLO, ROLR, and ROLO) and age (i.e., WOA 36 and 60) as well as their interaction. For each health parameter, models included focal hen ID nested in pen as a random term.

Production data were analyzed from 21 to 60 WOA and included: egg laying performance (%), feed consumption (kg), mortality (%), and floor eggs (%). Models included age as a continuous variable and treatment as well as their interaction as fixed factors. Pen was included as a random factor. To analyze laying performance, age was included in the model as a squared variable due to the parabolic model estimation. The response variables percentage of floor eggs and mortality were log transformed and raised to the third power, respectively, to achieve a normal data distribution.

All data and associated code are available www.doi.org/10.17605/OSF.IO/NBPXV.

## RESULTS

### Behavior

**Figure 2:**
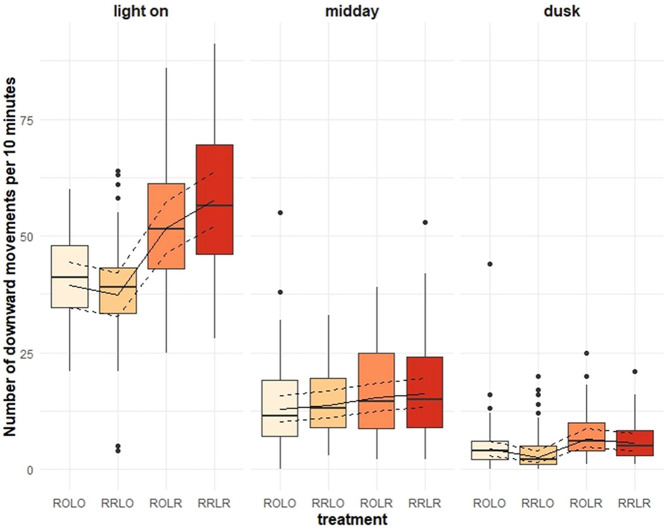
Effect of ramp provision and time of day on the number of downward transitions (*p* = 0.001). Ramp treatment groups were: no ramps (**ROLO**), ramps only during rearing (**RRLO**), ramps only during laying (**ROLR**) and ramps during both phases (**RRLR**). Raw data are represented with boxplots. The solid line represents the estimated means and the dashed lines the 95% confidence intervals of the full model.

**Figure 3:**
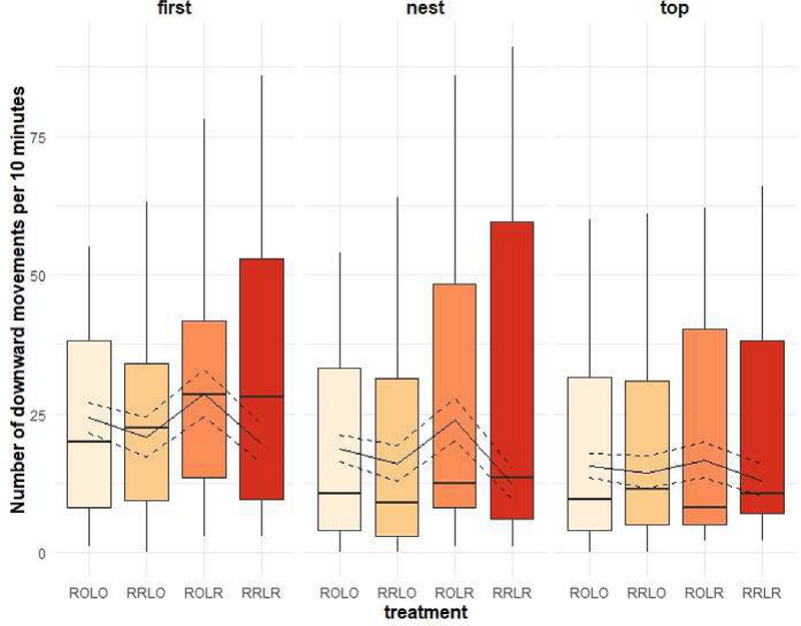
Effect of ramp provision and aviary tier on the number of downward transitions (*p* = 0.013). Ramp treatment groups were: no ramps (**ROLO**), ramps only during rearing (**RRLO**), ramps only during laying (**ROLR**) and ramps during both phases (**RRLR**). Raw data are represented with boxplots. The solid line represents the estimated means and the dashed lines the 95% confidence intervals of the full model.

The number of downward transitions was associated with the interaction of treatment and time of day (Figure 2, *p* = 0.001) where more movements were observed after lights on in groups that had ramps in both phases (model estimates and 95% confidence intervals for RRLR: 57.8 [52.0, 63.8]) or lay only (ROLR: 51.6 [46.2, 57.3]) compared to groups that did not (ROLO: 39.4 [34.7, 44.4] and RRLO: 37.3 [32.7, 42.2]). Differences in the number of downward movements were weak during the dusk phase (RRLR: 5.7 [4.0, 7.7], ROLR: 6.5 [4.7, 8.7], ROLO: 4.5 [2.9, 6.1], RRLO: 2.5 [1.5, 3.9]) and at mid-day (RRLR: 16.3 [13.3, 19.6], ROLR: 15.4 [12.5, 18.5], ROLO: 12.8 [10.2, 15.7], RRLO: 13.7 [11.0, 16.7]). Downward transitions were also affected by the tier from which hens began a transition and treatment (Figure 3, *p* = 0.013) as more downward transitions were performed from the first tier when birds were provided with ramps during both phases (model estimates and 95% confidence interval for RRLR: 26.6 [22.7, 30.7]) or in the laying phase only (ROLR: 26.3 [22.5, 30.5]) compared to birds that did not have ramps at any phase (ROLO: 19.5 [16.3, 23.1]) or during the rearing phase only (RRLO: 19.2 [15.9, 22.7]). The same pattern was found for downward transitions from the nest tier (RRLR: 22.5 [19.0, 26.3], ROLR: 21.8 [18.3, 25.5], ROLO: 14.9 [12.0, 18.0], RRLO: 12.0 [9.4, 14.8]) but did not exist for downward transitions from the top tier (RRLR: 17.0 [14.0, 20.3], ROLR: 14.9 [12.1, 18.1], ROLO: 13.4 [10.7, 16.4], RRLO: 12.7 [10.1, 15.6]). Age did not affect the number of downward transitions (*p* = 0.162).

In the pens where ramps were available, birds used them for most of the downward transitions between the aviary tiers. From all downward transitions counted throughout the experiment in the pens with ramps, 79% and 86% were performed with ramps for ROLR and RRLR groups, respectively. A detailed description of ramp use during downward transitions including the factors TOD and age is provided in Table 1.

**Table 1:** Ramp use (%) for downward transitions presented for the 2 treatment groups where ramps were provisioned during both (**RRLR**) or the laying phase only (**ROLR**) for both ages and time of day. For each time of day downward transitions were observed for 10 min and the number of observed downward transitions recorded separately per tier. Numbers in parentheses show the total number of downward movements observed per timepoint, time of day and aviary tier.

	19/20 WOA			30/31 WOA)		
	Lights on	Midday	Dusk phase	Lights on	Midday	Dusk phase
ROLR						
Top tier	77.6 ± 9.6 (393)	58.8 ± 15.7 (97)	88.9 ±14.7 (54)	75.6 ± 8.3 (312)	79.5 ± 16.8 (39)	96.8 ± 7.0 (31)
Nest tier	70.7 ± 13.0 (518)	63.4 ± 18.0 (142)	78.8 ± 17.1 (52)	69.2 ± 10.2 (468)	89.4 ± 11.5 (94)	85.1 ± 19.0 (47)
First tier	83.5 ± 3.8 (400)	85.4 ± 2.7 (246)	86.6 ± 15.4 (82)	89.4 ± 3.2 (425)	92.7 ± 5.7 (199)	86.4 ± 8.6 (81)
RRLR						
Top tier	88.2 ± 5.1 (382)	85.4 ± 14.6 (96)	89.9 ± 12.4 (79)	88.0 ± 2.5 (342)	85.9 ± 9.1 (64)	94.9 ± 11.8 (39)
Nest tier	80.7 ± 4.4 (548)	79.6 ± 12.0 (162)	83.7 ± 23.1 (49)	82.7 ± 6.5 (588)	86.2 ± 18.2 (94)	90.5 ± 35.1 (21)
First tier	86.6 ± 6.3 (485)	85.5 ± 10.9 (228)	89.5 ± 8.1 (76)	90.2 ± 5.2 (478)	91.7 ± 8.1 (216)	87.2 ± 21.5 (39)

Overall, 162 falls were observed during the video analysis with the majority of falls occurring during the dusk phase at both ages (19/20 WOA: 43 (26%) falls and 30/31 WOA: 80 (49%) falls; overall 75% of all observed falls). The remaining falls (39 falls) were observed during the lights on phase and no fall was observed at mid-day. Regarding treatments, the number of falls (19/20 WOA: 39.5% and 30/31 WOA: 60.5%) seemed to be less when more ramps were present: 19/20 WOA: RRLR = 8 falls (12.5 %), ROLR: 11 falls (17.2 %), RRLO = 27 falls (42.2 %) and ROLO = 18 falls (28.1 %); 30/31 WOA: RRLR = 16 falls (16.3 %), ROLR = 14 falls (14.3 %), RRLO = 29 falls (29.6 %), ROLO = 39 falls (39.8 %).

### Health Assessment

**Figure 4:**
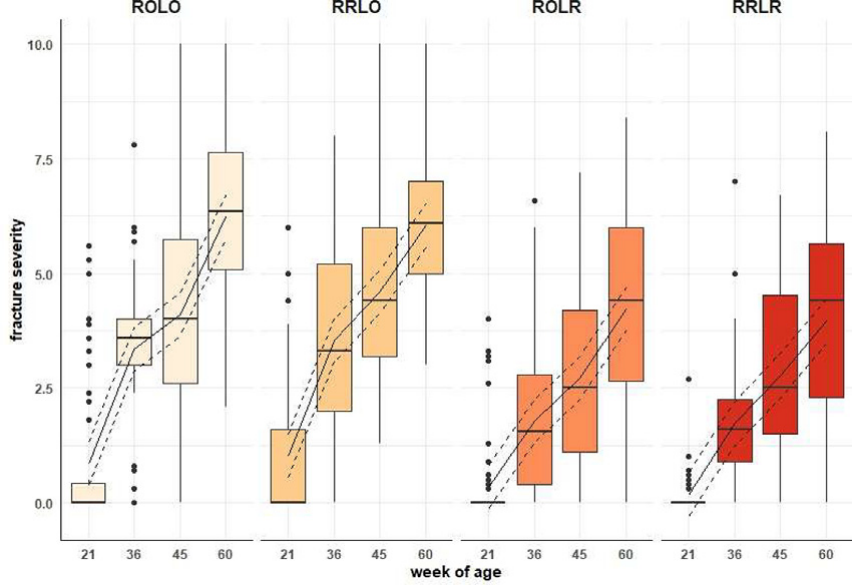
Effect of ramp provision week of age on the severity of keel bone fractures (*p* < 0.001). Ramp treatment groups were: no ramps (**ROLO**), ramps only during rearing (**RRLO**), ramps only during laying (**ROLR**) and ramps during both phases (**RRLR**). Raw data are represented with boxplots. The solid line represents the estimated means and the dashed lines the 95% confidence intervals of the full model.

***Keel Fracture Severity.*** Hens housed with ramps in lay (i.e., both RRLR and ROLR) had less severe KBF than those without ramps in the laying phase (*p* < 0.001). When provided ramps in the laying phase only (**ROLR**), the provision of ramps during rearing (**RRLO**) did not appear to affect severity of KBF. Differences in KBF severity only occurred among treatments after 21 WOA (Figure 4).

***Plumage Condition****.* The average feather score was affected by an interaction between treatment and age (*p* < 0.001). Overall, feather scores worsened over time (36 WOA: 9.2 ± 0.6 vs. 60 WOA: 7.8 ± 0.9) but feather scores were better at 60 WOA in hens that had access to ramps both in the rearing and laying phases (**RRLR**) compared to hens from the other treatment combinations (RRLR: 8.5 ± 0.7 vs. RRLO: 7.5 ± 0.7, ROLR: 7.4 ± 0.9, ROLO: 7.8 ± 0.9).

***Foot Health.*** Pododermatitis was affected by treatment (*p* = 0.018) with birds that had access to ramps during rearing as well as the laying phase (RRLR) being less likely to have pododermatitis compared with birds that never had access to ramps (RRLR: 0.11 ± 0.3 vs. ROLO: 0.26 ± 0.4) or with birds that had ramps only during rearing (RRLR: 0.11 ± 0.3 vs. RRLO: 0.23 ± 0.4). Foot health did not differ between the RRLR and ROLR treatments (RRLR: 0.11 ± 0.3 vs. RRLO: 0.18 ± 0.4).

***Body Mass.*** Body mass did not differ between treatment groups or ages (36 WOA: 1.698 kg ± 0.12; 60 WOA: 1.701 kg ± 0.14).

### Production Data

Egg laying performance, feed consumption per bird, and percentage of floor eggs (all expressed per live hen) were linked to age with more total eggs laid (*p* < 0.001), less feed consumed per bird (*p* < 0.001), and fewer floor eggs (*p* = 0.029) with increasing age. For example, at 24 WOA, laying performance (i.e., number of eggs/ hen/ day) was 82.6% compared to 94.9 % in WOA 60. Peak egg production was reached at 36 WOA with a laying performance of 97.9%. However, egg-laying performance, feed consumption per bird, and percentage of floor eggs did not differ among treatments. Mortality did not differ between treatments or by age. A summary of all production data is provided in Table 2.

**Table 2:** Production data presented as means ± SD for laying performance (%), feed consumption (kg), accumulated mortality (%) and floor eggs (%) collected from 18 to 60 wk of age for the 4 different treatment groups. Age affected laying performance and feed consumption (both *p* < 0.001) and percentage of floor eggs (*p* = 0.03). Treatment did not affect any of the production parameter (*p* > 0.05). Mortality was not affected by neither age nor treatment (*p* > 0.05).

	Week of age^1^												
Parameter	Treatment^2^												
		21	24	28	32	36	40	44	48	52	56	60	
Average laying performance (%)	ROLO	6.8 ± 0.1	81.6 ± 1.0	96.4 ± 0.7	97.8 ± 0.4	98.2 ± 0.8	98.4 ± 1.0	98.0 ± 0.7	97.5 ± 0.7	96.4 ± 0.4	95.6 ± 0.3	95.4 ± 0.7	
	ROLR	8.4 ± 1.8	83.8 ± 2.2	96.5 ± 0.6	97.7 ± 0.6	97.7 ± 0.6	97.8 ± 0.5	97.3 ± 0.6	97.1 ± 0.7	96.3 ± 1.1	95.2 ± 0.8	95.2 ± 0.2	
	RRLO	7.8 ± 3.0		82.1 ± 3.3	96.4 ± 1.4	97.2 ± 1.4	97.5 ± 0.9	97.6 ± 1.0	97.0 ± 1.5	96.1 ± 1.3	96.0 ± 0.9	94.9 ± 0.8	94.5 ± 1.2
	RRLR	7.2 ± 3.6		83.0 ± 3.6	96.5 ± 0.2	97.3 ± 0.2	97.9 ± 0.6	98.0 ± 1.4	97.2 ± 0.8	97.6 ± 0.6	96.4 ± 1.2	95.5 ± 1.3	94.5 ± 0.8
Average feed consumption (kg)	ROLO	NA		2.35 ± 0.07	2.12 ± 0.05	2.07 ± 0.03	1.97 ± 0.04	1.94 ± 0.05	1.88 ± 0.03	1.86 ± 0.02	1.88 ± 0.01	1.89 ± 0.01	1.85 ± 0.02
	ROLR	NA		2.34 ± 0.02	1.99 ± 0.06	2.06 ± 0.03	2.0 ± 0.04	1.96 ± 0.03	1.90 ± 0.00	1.88 ± 0.02	1.88 ± 0.02	1.91 ± 0.03	1.82 ± 0.05
	RRLO	NA		2.34 ± 0.13	2.05 ± 0.14	2.08 ± 0.02	1.97 ± 0.03	1.95 ± 0.03	1.89 ± 0.03	1.87 ± 0.03	1.89 ± 0.03	1.91 ± 0.02	1.84 ± 0.01
	RRLR	NA		2.34 ± 0.07	1.98 ± 0.06	2.08 ± 0.02	1.99 ± 0.03	1.94 ± 0.02	1.90 ± 0.01	1.85 ± 0.01	1.89 ± 0.02	1.85 ± 0.01	1.86 ± 0.00
Accumulated average mortality (%)	ROLO	0 ± 0.0		0.33 ± 0.7	0.22 ± 0.3	0.33 ± 0.7	0.11 ± 0.2	0.22 ± 0.4	0.22 ± 0.3	0 ± 0.0	0.44 ± 0.6	0.11 ± 0.2	0.45 ± 0.6
	ROLR	0.2 ± 0.2		0.22 ± 0.3	0.22 ± 0.3	0 ± 0.0	0.22 ± 0.3	0 ± 0.0	0.11 ± 0.2	0.11 ± 0.2	0.55 ± 0.4	0.22 ± 0.3	0.56 ± 0.6
	RRLO	0 ± 0.0		0.33 ± 0.2	0.33 ± 0.4	0.11 ± 0.2	0.33 ± 0.4	0.22 ± 0.4	0 ± 0.0	0.11 ± 0.2	0.11 ± 0.2	0.22 ± 0.3	0.22 ± 0.3
	RRLR	0 ± 0.0		0.33 ± 0.2	0.22 ± 0.4	0 ± 0.0	0.11 ± 0.2	0.33 ± 0.3	0.11 ± 0.2	0.11 ± 0.2	0.22 ± 0.3	0.11 ± 0.2	0.33 ± 0.4
Average number of floor eggs (%)	ROLO	0.25 ± 0.2		0.87 ± 0.2	0.19 ± 0.2	0.06 ± 0.1	0.10 ± 0.2	0.13 ± 0.2	0.11 ± 0.2	0.15 ± 0.2	0.15 ± 0.2	0.13 ± 0.1	0.22 ± 0.2
	ROLR	0 ± 0.0		0.98 ± 0.3	0.41 ± 0.3	0.35 ± 0.3	0.10 ± 0.1	0.04 ± 0.0	0.07 ± 0.0	0.03 ± 0.0	0.07 ± 0.0	0.18 ± 0.1	0.20 ± 0.2
	RRLO	0.16 ± 0.1		0.91 ± 0.4	0.08 ± 0.1	0.09 ± 0.1	0.05 ± 0.0	0.04 ± 0.0	0.06 ± 0.0	0.05 ± 0.0	0.12 ± 0.1	0.31 ± 0.3	0.35 ± 0.3
	RRLR	0.14 ± 0.2		1.03 ± 0.4	0.23 ± 0.2	0.15 ± 0.2	0.14 ± 0.2	0.09 ± 0.2	0.12 ± 0.2	0.11 ± 0.1	0.16 ± 0.2	0.2 ± 0.2	0.26 ± 0.2

1 Data on eggs laid, mortality and feed consumed was recorded per pen and day and incorporated the number of live hens on that day. Data were averaged across every 4 weeks of age, for example, the column for week of age 21 includes all values averaged from 18 to 21 weeks of age per treatment group.

2 The different treatment groups consisted of ROLO: no ramps at all, ROLR: ramps during laying phase only, RRLO: ramps during rearing phase only and RRLR: ramps during both phases. Each treatment group had 4 replicates in the laying barn.

## DISCUSSION

The current study builds on previous work from our group examining the benefits of ramps placed in rearing (Stratmann et al., 2022) by extending observations into the laying phase. Findings from these efforts, as well as those of others exploring the benefits of ramps during rearing within aviaries (Pettersson et al., 2017; Norman et al., 2018, 2021; Zheng et al., 2019), generally support positive short- and long-term outcomes for ramps on animal health and welfare. While the collective findings are encouraging, it is important that the benefits are clarified, including critical periods of use, specific mechanisms, and types of ramps that deliver the best overall outcome within particular housing systems. We believe the current study, by exploring provision or absence of ramps during both the rearing and laying phase within quasi-commercial conditions across a wide array of responses, provides information towards optimizing laying hen housing.

Observations in the laying pens that contained ramps (RRLR, ROLR) found that the vast majority of all downward transitions occurred using ramps rather than other means (e.g., jumping, flying) with an average of 82% or more per pen (ranging from 58.8 to 96.8% depending on tier and time point). We have previously reported comparable findings for transitions using ramps during rear (Stratmann et al., 2022) suggesting a genuine preference for using ramps to walk between tiers instead of jumping or flying. Our findings have important implications given the increased consideration of animals’ natural needs and motivations within animal welfare assessments and policy decisions. For instance, Swiss legislation mandates that housing does not infringe on the normal biological function of the animal (Art. 3, TSchV, Switzerland, 2008). If hens are to gain access to foraging and dust bathing substrates as well outdoor verandas or ranges, they must be able to navigate between the upper areas and floor (Pettersson et al., 2016). Hens must also be able to navigate to nests for laying eggs and higher tiers of the aviary for roosting at night (Campbell et al., 2016a;b; Villanueva et al., 2017; Ali et al., 2019). Access to resources may be compromised if vertical movement through the aviary is physically difficult for birds (Nasr et al., 2012; Rentsch et al., 2019; Rufener et al., 2020), inhibiting bird movement. We believe our findings provide a strong argument for incorporating ramps in aviary systems during both rear and lay ensuring access to these resources are maintained. Although many efforts have been made to determine what the bird's true motivations and needs are, the task is difficult, even whether the supposed ‘natural’ preferences are best for the animal's welfare (Dawkins, 2023). As an example of the difficulties in ensuring housing best fits the animals’ needs, the public typically associates laying hens’ use of the range with good welfare, though data from our group suggests a covered winter garden (that provides security and protection from weather) may actually be preferred by the majority of the flock (Gómez et al., 2022). From a behavioral standpoint, the current study functioned as an de facto preference test where birds chose overwhelmingly to transition with ramps. Chickens are terrestrial birds, preferring to walk or use wing-assisted-incline-running rather than flying when moving around their environment (Sandilands et al., 2009; Stratmann et al., 2015a). More critically, chickens are poorly suited for the guided, precision flight needed to descend from upper to lower areas within the aviary where open space is limited (Tobalske et al., 2003; Tobalske, 2007). Ramps that connect the tiers in aviary housing systems facilitate bipedal locomotion among vertical tiers, thus reducing or eliminating the necessity to fly or jump. Our findings of an overall preference to use ramps and reduced fracture severity, in combination with comparable findings of others, as well as an underlying biomechanical profile that fits vertical transitions with ramps justify the use of ramps within multi-tier aviaries throughout the animal's life.

Beyond the results mentioned above, there are also more subtle findings which may inform how and where ramps are best placed. Although ramps will provide a useful path to transition to the floor area from the upper tier following lights coming on and the evening roost, there was also a substantial number of downward transitions from the first tier to the floor involving ramps. While descents between the upper tier and nest are complicated (involving landing on a perch), and descents to the floor are long (3.0 meters) and thus dangerous, transitions from the first tier to the floor seem relatively innocuous being short distances with a landing area covered in litter. Nonetheless, the birds still used the ramps for a large number and percentage of these transitions. It is likely that most birds remain in the lower tiers after lights come on, then access the first tier for fresh feed and water resulting in many transitions into the litter area, evidence supported by tracking of individual hens (Rufener et al., 2018c; Sibanda et al., 2020; Montalcini et al., 2023).

In considering the benefits of providing ramps during rearing, there did not seem to be a major effect on later KBF which was surprising given the expected cognitive/navigation/strength benefits (Tahamtani et al., 2015; Norman et al., 2019). The rearing aviaries had multiple tiers and all had ramps onto the litter from the first tier (even in the RO treatment) which may have provided adequate exposure to variable heights of structures and ramps for benefits in lay to be realized independent of treatment. Nonetheless, as discussed above, ramps may provide additional benefits beyond KBF in assessing appropriate housing designs. For instance, the proportion of downward transitions across location and TOD in pens with ramps during lay were more consistent and greater when birds also had ramps in rear (RRLR) compared to those that did not (ROLR). The range of ROLR values was 30.1 and 27.6 at 19/21 and 30/31 WOA, respectively, whereas the range of RRLR values (10.3 and 12.2, respectively) was approximately a third of those values. Although not statistically evaluated, the consistently greater overall usage of the RRLR pens could suggest that the exposure during rearing allowed for more familiarity with ramps. Lastly, the initially higher numeric output of falls in RRLO in the first time point (i.e., 27) may indicate the birds were having difficulty in transitioning to ramps not being available in the laying phase. In either case, ramps in lay did seem to reduce falls and collisions.

Ramps were also associated with other improvements to health in the form of feather quality and footpad health. Regarding feather quality, increased daytime litter use by hens has been linked with better plumage quality (Ali et al., 2020) and RRLR hens transitioned more to the litter in the period up to 30 WOA. If the RRLR hens spent more on the litter overall, it may explain the improved feather score at 60 WOA, though future efforts will need to examine where hens actually remain over the course of the day. Dust bathing on litter also results in healthier plumage, via removal of lipids and ectoparasites (van Liere, 1992; Sandilands, 2001). As litter use also promotes foraging, there may also be benefits to reduce feather pecking (see Rodenburg et al. (2013) for a review). Better foot health was also linked with RRLR hens while birds who either never had access to ramps or did not have access during lay had the worst scores. These results are consistent with those of Heerkens et al. (2016) who also found that providing ramps to hens in aviaries reduced prevalence of pododermatitis. Improved foot health seen by birds with ramps may be a consequence of their ease in accessing the litter. Similar to the scenario for feather coverage, if the RRLR hens spent more time on the litter with increasing age (up to 36 WOA) it could explain our findings. More frequent daytime use of litter areas by hens was found by Ali et al. (2020) to be associated with lower odds of pododermatitis which supports this explanation. Another possibility suggested by Heerkens et al. (2016) is that the wire structure of the ramps might improve foot cleanliness by scraping manure from the foot pads, improving hygiene and reducing potential for pododermatitis (Wang et al., 1998). In support of this idea, Tauson and Abrahamsson (1994) reported a lower incidence of bumble foot in hens that had wire platforms installed as perch space compared to hens without such wire platforms.

Our study also allowed for a robust analysis of production endpoints. Although age effects were as expected in that no treatment effects were found, we believe this should be interpreted positively in light of ramps having clear benefits in terms of the hens’ overt preferences and multiple health indicators. If providing ramps is not associated with production detriments, the only real downside is the cost of installation. Our ramp installation was fairly simple and, especially in the laying barn where each pen is relatively narrow (2.3 m), is not applicable to true commercial conditions. However, despite the cost concerns, industry has been adapting their products to reflect the interest and perceived benefits of ramps. For instance, one major manufacturer of laying hen aviary systems now offers ramps as a standard option where they estimate that ramp installation in a Bolegg Terrace Aviary system (as used in the current study) at every second or third section would increase costs by 2.5 to 3.5% (personal communication, B Liebregts), an amount that seem to justify the expense in light of benefits.

In conclusion, our results reinforce previous findings that providing ramps during the lay period increases movement among tiers and improves animal health including pododermatitis, plumage, and keel fractures. Although ramps at rearing did not appear to reduce keel fractures in laying hens, the presence of ramps to the floor from the first tier in all rearing aviaries may have stimulated sufficient development of bone, muscle, and navigational skills that was not further enhanced by adding ramps throughout the rest of the rearing aviary. Providing ramps during the lay period did not negatively impact hen health or productivity. These findings add to the scientific understanding of managing laying hens in aviaries that can be used by industry stakeholders and industrial manufacturers to provide options for improving structural design of rearing and laying aviaries. Our findings also further our understanding of factors important to consider during transfer between rearing and laying environments to facilitate adaptation to the new environment and as a consequence, improve general quality of life for laying hens in aviaries.

## DISCLOSURES

The authors declare no conflicts of interest.

## Appendix Supplementary materials

Image, application 1
