# Supplementary material for: Providing ramps during lay has larger impacts on laying hens than ramps at rearing
Source: Poult Sci. 2024 Jul 31;103(10):104101. doi: 10.1016/j.psj.2024.104101 (PMC11372577; doi:10.1016/j.psj.2024.104101)
Supplement: Supplementary file 1 [file mmc1.docx]

|  |  | Chick starter | Lay |
| --- | --- | --- | --- |
| Energy | MJ | 11.8 | 11.7 |
| Protein | % | 17.7 | 17.5 |
| Fiber | % | 5.7 | 4.5 |
| Lysin | % | 0.8 | 0.81 |
| Methionin | % | 0.17 | 0.47 |
| Calcium | % | 1.2 | 3.6 |
| Phosphorous | % | 0.27 | 0.48 |
| Sodium | % | 0.36 | 0.15 |
| Magnesium | % | 0.28 | 0.15 |
